# An integrative genomics approach for mapping the emerging genetic susceptibility landscape of triple-negative breast cancer

**DOI:** 10.1186/1753-6561-6-S6-P14

**Published:** 2012-10-01

**Authors:** Chindo Hicks, Antonio Pannuti, Kandis Backus, Lucio Miele

**Affiliations:** 1 Cancer Institute, University of Mississippi Medical Center, 2500 N. State Street, Jackson, MS 39216, USA

## Background

Genome-wide association studies (GWAS) have achieved great success in identifying common genetics variants associated with increased risk for developing breast cancer. More recently, advances in next-generation sequencing (NGS) have made possible identification of mutations associated with breast cancer. However, to date, the information generated by GWAS and NGS has not been maximally leveraged and integrated with gene expression data to identify biomarkers associated with the most aggressive subset of breast cancer: the triple-negative breast cancer (TNBC). Here we present results from an integrative genomics approach that combines GWAS and sequence information with gene expression data to identify functionally related genes and biological pathways enriched for expression-associated genetic loci and mutations associated with TNBC using publicly available data.

## Material and methods

We used publicly available data derived from 60 GWAS involving over 400,000 cases and over 400,000 cancer-free controls to identify SNPs and associated genes with increased risk of developing breast cancer. Specifically, we first identified SNPs in population-based human cohorts that are associated with the expression of genes in TNBC. Mutations and associated genes were identified by mining publicly available RNA-Seq and whole exome/ genome sequencing data derived from 104 TNBC patients. Gene expression data were from 124 TNBC tumors and 142 cancer-free controls. We performed supervised and unsupervised analysis on gene expression data from genes containing genetic variants and mutations to identify functionally related genes. Additionally, we performed pathway prediction and network modeling using Ingenuity. For each predicted pathway and network, we counted the number of SNPs and mutation events by direct enumeration.

## Results

We identified 600 SNPs mapped to 205 genes, and 250 genes with mutations that included inserts, deletions (Indels) and copy number variants. Hierarchical clustering revealed functional relationships and similarity in patterns of expression profiles between SNP-containing genes and genes containing mutations. We identified multi-gene biological pathways enriched for SNPs and mutations. Many of the pathways identified have been proposed as important candidate pathways for TNBC, including the p53, NFkB, apoptosis, BRCA, DNA repair and DNA mismatch repair pathways.

## Conclusion

The results provide convincing evidence that integrating GWAS and sequence information with gene expression data provides a unified and powerful approach for biomarker discovery in TNBC. Furthermore, the results provide insights about the broader context in which genetic variants and mutations operate in TNBC.

